# Neurocognitive Trajectories of Scalar Implicature in Mandarin-Speaking Children: ERP Evidence for Attentional Allocation and Pragmatic Recalibration (4–6 Years)

**DOI:** 10.3390/bs16030371

**Published:** 2026-03-05

**Authors:** Lulu Cheng, Wenting Yuan, Haoran Mao, Yule Peng, Lei Jia, Bingqi Fu, Xize Jia

**Affiliations:** 1 School of Foreign Studies, China University of Petroleum (East China), Qingdao 266580, China; 2 Faculty of Western Languages, Heilongjiang University, Harbin 150080, China; yuanwenting@hiu.edu.cn; 3 College of Foreign Languages, Ocean University of China, Qingdao 266100, China; pengyule@ouc.edu.cn; 4 School of Psychology, Zhejiang Normal University, Jinhua 321004, China

**Keywords:** SI, ERP, contextual influences, Mandarin-speaking children

## Abstract

Despite the centrality of scalar implicature (SI) in pragmatic development, the neurocognitive trajectory of SI processing in Mandarin-speaking children remains underexplored, with existing frameworks inadequately accounting for developmental constraints and cross-linguistic variation. This ERP study maps the neurocognitive trajectory of scalar implicature (SI) processing in Mandarin preschoolers (N = 49). Behavioral accuracy improved with age (*p* < 0.001) but was not modulated by contextual felicity. Neural dynamics revealed developmental shifts: 4-year-olds exhibited heightened P200 amplitudes in infelicitous contexts, indicating attentional overloading. Differences in P200 amplitude between younger and older children indexed developmental shifts in attentional allocation. The N400 showed contextual sensitivity, whereas the Late Positive Component (LPC) showed only marginal context effects, suggesting protracted inferential adjustments. We propose the Cognitive-Dynamic Relevance Model (CDRM), challenging existing frameworks by integrating gradual recalibration mechanisms with resource constraints. Mandarin children demonstrate delayed SI maturation, attributable to reduced SI frequency in child-directed speech and quantifier ambiguity. Findings underscore cross-linguistic variation in pragmatic development, with neurocognitive markers preceding behavioral mastery.

## 1. Introduction

Scalar implicature (SI) explains how individuals infer implied meanings in communication, requiring an interplay of semantic knowledge, pragmatic reasoning, and cognitive control. SI involves deriving an upper-bounded pragmatic enrichment (e.g., “some but not all”) from a lower-bounded semantic meaning, a process that is sensitive to context and cognitive resources. Aligned with *China’s national Guidelines for the Learning and Development of Children Aged 3–6* (2012), a national policy document outlining developmental expectations for preschoolers in the language domain are expected to achieve core competencies encompassing both receptive (“listen attentively and comprehend everyday language”) and expressive (“speak willingly and express themselves clearly”) communication. This progression entails a developmental shift from literal meaning comprehension to contextually appropriate pragmatic interpretation. Thus, navigating the semantic–pragmatic interplay represents a key milestone in early language development. Nevertheless, the neurocognitive trajectory underpinning SI acquisition remains contentious and underexplored, particularly in non-Western languages. Empirical findings present a complex picture. Noveck and Posada (2003) found that children favor semantic interpretations, whereas Panizza et al. (2021) showed that 3- to 4-year-olds successfully derive pragmatic SI under enriched contexts. These inconsistencies highlight the potential influence of cross-linguistic factors, which existing theoretical accounts often fail to incorporate adequately.

Mandarin Chinese thus presents a theoretically informative and challenging case. The pragmatic enrichment of “yixie” necessitates heavier reliance on contextual information, potentially altering both the developmental trajectory and the cognitive demands of SI processing (S. Zhao et al., 2021). Based on both prior corpus-based research and our own frequency counts conducted on the CHILDES child-directed speech corpora, the Mandarin quantifier “yixie” occurs substantially less frequently than its English counterpart “some”. Our CHILDES-based analysis indicates that “yixie” appears at approximately 0.8 occurrences per 1000 utterances in Mandarin child-directed speech, whereas “some” appears at approximately 2.3 occurrences per 1000 utterances in English child-directed speech (CHILDES-Zhou Dinner; EDINBURGH, https://childes.talkbank.org). These estimates are consistent with previously reported corpus findings (Wu, 2023; Eiteljoerge et al., 2018). This lower distributional frequency, together with the semantic underspecification of “yixie” (which is compatible with universal constructions such as “yixie…dou”), likely increases children’s reliance on contextual and cognitive resources for pragmatic enrichment. Neurocognitive findings reinforce this hypothesis, revealing language-specific neural patterns, such as prolonged N400 latency (300–500 ms) during Mandarin SI processing compared to Germanic languages (Panizza et al., 2021: 250–400 ms), suggesting a delayed or more effortful pragmatic integration (S. Zhao et al., 2021).

These cross-linguistic divergences and developmental puzzles expose significant gaps in current theoretical models. Dominant accounts, including neo-Gricean default theories and post-Gricean contextualist approaches (e.g., Relevance Theory), primarily offer synchronic explanations of adult processing. They inadequately capture the dynamic, resource-constrained, and gradual nature of pragmatic development in children. A framework that integrates diachronic change with real-time cognitive processing is therefore urgently needed.

To address this theoretical and empirical gap, the present study employs event-related potentials (ERPs) to investigate the neurocognitive trajectory of SI in Mandarin-speaking preschoolers aged 4–6 years. We track developmental shifts in neural correlates, specifically the P200 (attentional allocation), N400 (semantic memory), and LPC (pragmatic re-evaluation) associated with SI processing under felicitous and infelicitous contextual conditions. On the basis of our findings, we propose the Cognitive-Dynamic Relevance Model (CDRM), which extends existing frameworks by formally incorporating principles of diachronic equilibrium and resource-constrained optimization. The CDRM provides a more nuanced account of how pragmatic inferencing gradually recalibrates and automatizes during the preschool years.

By elucidating the neurocognitive underpinnings of SI development in Mandarin, this study not only challenges universalist assumptions about pragmatic acquisition but also advances a refined, developmentally theoretical model. Our work has broader implications for understanding the interplay between language, cognition, and culture in early childhood.

## 2. Literature Review

### 2.1. Empirical Research on Children’s SI Processing

The empirical investigation of scalar implicature (SI) processing in children has progressively advanced from behavioral paradigms to incorporating neurophysiological techniques, yet neural-level evidence remains limited. Noveck (2001) pioneered this empirical exploration by investigating SI processing in children aged 5–9 years and adults. This groundbreaking research garnered significant attention in linguistics and cognitive science, fostering interdisciplinary collaboration and deepening the academic understanding of language use, meaning derivation, and their underlying psychological mechanisms. To date, research on children’s SI processing has predominantly relied on behavioral experiments, while studies utilizing eye-tracking and EEG remain limited. These emerging techniques hold the potential to provide more nuanced insights into cognitive and neural mechanisms underlying children’s interpretation of SI.

Behavioral research predominantly employs truth-value judgment, aiming to analyze the developmental characteristics of children’s SI acquisition. Additionally, some researchers have introduced real-life contexts by employing picture-matching tasks, story scenarios, dialogues to further investigate the nature of children’s SI processing. Experimental results indicate that children often favor logical or semantic interpretations of scalar alternatives (Papafragou & Tantalou, 2004; Noveck, 2001; Teresa Guasti et al., 2005; Pouscoulous et al., 2007). Until the age of five, children typically struggle to infer the pragmatic meaning implied by “some” (Foppolo et al., 2012). The protracted pragmatic development observed here aligns with the ‘pragmatic tolerance hypothesis’ (Katsos & Bishop, 2011), wherein children prioritize logical meanings until sufficient cognitive resources enable context-driven SI derivation.

However, some researchers have improved experimental designs by presenting test content in dialogue formats to provide participants with richer contextual information, which demonstrated that children exhibit sensitivity to scalar terms and show emerging pragmatic reasoning abilities for SI, despite significant differences in performance compared to adults (Noveck, 2001; Papafragou & Tantalou, 2004; Katsos & Bishop, 2011). Notably, although short-term task-based training and experimental scaffolding can temporarily enhance children’s metapragmatic performance (Papafragou & Musolino, 2003), longitudinal evidence suggests that these gains are often not fully sustained over time (Teresa Guasti et al., 2005). This pattern indicates that metapragmatic competence is not solely training-driven but reflects a protracted developmental process constrained by broader cognitive and inferential maturation.

In addition, only a limited number of studies have utilized ERPs to examine children’s SI processing, but these provide valuable insights into the dynamic cognitive and neural mechanisms involved. The P200, an early positive deflection typically peaking around 150–250 ms, is commonly associated with early attentional allocation and perceptual categorization (Potts et al., 2004; Kandel et al., 2010). In language tasks, it has also been linked to early lexical activation and contextual anticipation (Freunberger et al., 2007). In SI paradigms, P200 effects are interpreted as reflecting the initial mobilization of attentional resources when listeners encounter contextually constrained scalar expressions (S. Zhao et al., 2021). The language-related N400 component, typically distributed over central and parietal electrodes, is widely associated with lexical–semantic access and semantic–contextual integration, and is reliably elicited when linguistic input violates semantic expectations (Kutas & Federmeier, 2011). Noveck and Posada’s (2003) study on adults showed that N400 amplitudes elicited by pragmatically under-informative sentences containing “some” were smaller than those elicited by semantically anomalous sentences. Nieuwland et al. (2010) further demonstrated that individuals with higher pragmatic competence exhibited larger N400 responses when processing pragmatically under-informative sentences, indicating greater effort in semantic–contextual integration under communicative uncertainty. Later positive components, including the P600 and the Late Positive Component (LPC), are more commonly associated with late-stage reanalysis, inferential adjustment, and integrative updating processes. While the P600 was initially linked to syntactic restructuring, many language and pragmatics studies interpret P600/LPC effects more broadly as reflecting reanalysis and interpretive revision. In SI research, these late positivities are often taken to index pragmatic reevaluation, conflict resolution, and memory-supported inferential processing. LPC effects may partially overlap with P600 in both timing and functional interpretation. While adults may exhibit automatized SI derivation (Levinson, 2000), children’s ERP markers (e.g., sustained N400 and LPC—the Late Positive Component, a late positive-going waveform commonly associated with pragmatic reevaluation and memory integration processes, and partially overlapping in latency and function with the P600) suggest effortful, resource-dependent computation shows a more resource-dependent processing profile. More recently, Panizza et al. (2021) used ERPs to compare German-speaking children aged 3–4 years and adults when interpreting the scalar term “some” under felicitous and infelicitous contexts, confirming that even young children can derive pragmatic SI under supportive contextual conditions. Cross-linguistic studies reveal that Mandarin quantifiers exhibit higher semantic ambiguity compared to English quantifiers, necessitating greater contextual integration for pragmatic inference. Armstrong et al. (2018) demonstrated in an ERP study that Mandarin-speaking children elicited larger P600 when processing ungrammatical sentences when the quantifier was absent compared to English peers, reflecting increased cognitive effort in resolving its context-dependent interpretations. This aligns with findings that Mandarin SI processing involves stronger reliance on quantificational cues and alternative-based generation ability for pragmatic enrichment (S. Zhao et al., 2021), underscoring language-specific neurocognitive dynamics shaped by lexical–semantic granularity and cultural communicative norms. However, several important questions remain open after these contributions. Existing Mandarin ERP studies, including S. Zhao et al. (2021), have primarily focused on adult participants and have mainly established the presence of language-specific neural responses associated with scalar implicature processing. What remains unclear is how these neural mechanisms develop across early childhood, and how SI-related neural dynamics reorganize across successive developmental stages. In particular, prior work has not systematically examined age-related trajectories in preschool populations, nor has it provided a process-level developmental account linking attentional allocation (P200), semantic integration (N400), and late pragmatic reevaluation (LPC/P600). Addressing these unresolved issues requires a developmentally targeted ERP investigation, which the present study is designed to provide.

It is evident that different methods have yet to achieve a consensus on the empirical findings related to SI, and notable disparities exist in meaning interpretation of SI. To address these discrepancies, some scholars have investigated the factors that constrain SI processing, focusing on working memory capacity and executive control functions, personality-based pragmatic abilities, and specific contextual factors, including politeness principles, face theory, prosody, and intonation (Nieuwland et al., 2010; M. Zhao et al., 2015; Yang et al., 2018; Holtgraves & Kraus, 2018; Huang & Snedeker, 2018; Tomlinson et al., 2017). While the factors influencing children’s processing of SI remain inconclusive, SI processing solely reflects the hearer’s pragmatic inferencing of the speaker’s intentions. This not only highlights the complex psychological and cognitive processes underlying human language comprehension but also provides a valuable perspective for advancing research on children’s pragmatic development.

### 2.2. The Relationship Between Children’s SI Inferencing and Their Developmental Pragmatic Ability

Different pragmatic schools focus primarily on the issue of SI’s meaning attribution, centering on the interactive relationship between the logical and its communicative meaning, exploring the speaker’s psychological intentionality, specifically the inference of communicative intentions. The process of inferring communicative intentions is a manifestation of rational behavior in humans, with early sensitivity to communicative agents developing in the initial stages of language acquisition (Tomasello, 2018; Frank & Goodman, 2012). Children’s SI inferencing is the result of social inferences about the speaker’s communicative intention, based on the communicator’s knowledge, discourse goals, and other mental states (Kampa & Papafragou, 2023). SI derivation is constrained by expectations of rational behavior, which requires children to further infer the speaker’s state of knowledge, following social communicative norms. Post-Gricean school’s meaning interpretation model guided by relevance theory seeks to explain how listeners infer the speaker’s meaning based on linguistic evidence and available contextual information. Pragmatic reasoning, characterized by systematicity and robustness, is universally present in daily human communication and reflects rational communicative behavior. Current research on pragmatic reasoning mainly focuses on non-literal meaning expressions such as SI, metaphor, irony, and metonymy (Falkum, 2022). The pragmatic inference process of SI begins with the linguistic encoded meaning of scalar alternatives, which is then expanded through pragmatic enrichment and integrated with contextual information, world knowledge, and speaker’s utterance goals. The semantic and pragmatic division model of SI highlights the stability and flexibility of human communication through language, but the mechanisms of meaning inference in language development remain insufficiently understood.

The psychological inference abilities is crucial to pragmatic development (Fairchild & Papafragou, 2021). Pragmatic development is closely related to cognitive ability (Cheng et al., 2024) and psychological inference ability, particularly the development of theory of mind (E. Bates, 1976). As children age, the growth of their cognitive abilities further enhances their capacity to infer implicatures. Given that SI involves the speaker’s inference of both semantic and pragmatic meaning, Horowitz and Frank (2015) identify the inferential process of SI as a key indicator for assessing children’s pragmatic developmental abilities.

In recent years, researchers have empirically examined the dialectical relationship between SI and psychological inference ability, noting that the successful acquisition of SI reflects the hearer’s capacity to infer the communicator’s epistemic state (Fairchild & Papafragou, 2021). During the Concrete Operational Stage, children aged 4 to 5 are already capable of adjusting discourse content from the perspective of others and inferring the epistemic states of others (De Villiers & de Villiers, 1979). Some scholars argue that the development of children’s pragmatic abilities is grounded in the development of metalinguistic competence, based on Grice’s Cooperative Principle and conversational maxims (Röhrig, 2010), which means children already possess the ability to encode and decode linguistic information during the concrete operational period. By the age of 4, children possess the reasoning ability to infer the speaker’s intentions (Wilson et al., 2020), but few researchers find that children under the age of 5 still struggle to understand the pragmatic content implied by sentences containing scalar alternatives (Noveck, 2001; Pouscoulous et al., 2007). Thus, an in-depth exploration of the derivation mechanisms of SI in children not only opens new directions for understanding meaning attribution in SI but also enriches research on children’s pragmatic developmental abilities.

It is evident that previous research on children’s SI processing faces several key limitations. First, most studies have focused on specific age groups or conducted comparative analyses between children and adults, lacking systematic investigations across different developmental stages. Second, the majority of research relies on traditional behavioral methods, with limited use of advanced and dynamic cognitive science techniques. Finally, existing empirical findings have yet to establish a unified theory of meaning interpretation, leaving the nature of children’s SI processing insufficiently explained.

### 2.3. Theoretical Development of SI

Building on Grice’s proposal, subsequent accounts diverge into Neo-Gricean default approaches and Post-Gricean contextualist approaches. The Neo-Gricean school, represented by Horn and Levinson, has proposed a revised interpretative model for SI, building on the classical Gricean theory. Horn extended the core principles of conversational implicature theory, addressing ambiguities and vagueness in earlier models. By integrating conversational maxims with a focus on linguistic forms, he emphasized their crucial role in implicature inference. Horn’s approach to scalar alternatives involves the interaction between the lower-bound (semantic content) and the upper-bound (pragmatic content) (Horn, 2009). However, Levinson (2000) proposed a default theory by dividing communicative content into “coded meaning” and “speaker’s meaning or implicature”. SI is generated automatically and is characterized by default properties, with its meaning often being canceled out in cases of contextual conflict. Therefore, the essential nature of SI cannot be fully attributed to either the semantic or the pragmatic domain. However, ERP evidence (Nieuwland et al., 2010; M. Zhao et al., 2015) suggests that SI derivation incurs cognitive effort, contradicting claims of automatic processing.

The post-Gricean school primarily includes two perspectives on the meaning attribution of SI: the context-driven view centered on relevance theory (Sperber & Wilson, 1995) and the semantic–pragmatic integration view based on default semantics theory (Jaszczolt, 2005). The former view emphasizes that SI is generated by the speaker in accordance with the relevance principle within a specific context, representing a holistic meaning processing model that relies on the felicitous context and requires the mobilization of cognitive processing resources. The latter view categorizes SI within the framework of default semantics, arguing that its derivation is influenced by pragmatic factors and belongs to a compositional merger representation model of meaning interpretation. By contrast, default semantics theory has not yet provided an explicit developmental account of how SI processing changes across age groups.

Various models for interpreting SI reveals that the core issue lies in the philosophical debate over the boundary between semantics and pragmatics. This divergence has led to differing theoretical perspectives on the essence of SI. To address these issues, Parikh (2010) proposed equilibrium semantics in his book *Language and Equilibrium*. Parikh incorporates game theory into the study of linguistic meaning, arguing that meaning selection is fundamentally a dynamic equilibrium process. It does not prioritize the sequence of propositions or utterance meaning but instead synchronizes sentence propositions with utterance meaning, achieving a balanced exchange of information between speaker and hearer during communication. As communication progresses, the listener’s decoding of information becomes a dynamic and evolving cognitive process. However, previous applications of this model have primarily focused on adult language use, without a clear developmental perspective.

The SI theoretical debate crystallizes in the default–contextualist divide: Levinson (2000) attributes SI to automatic pragmatic defaults, while Sperber and Wilson (1995) situate it in context-driven relevance optimization. Crucially, both frameworks inadequately address developmental trajectories and cognitive constraints in children’s SI acquisition.

### 2.4. The Cognitive-Dynamic Relevance Model (CDRM)

**Figure 1:**
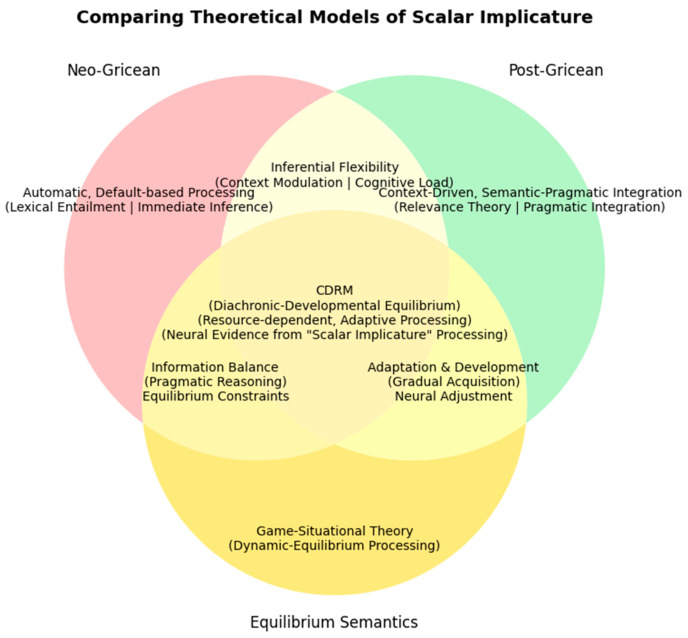
Comparing Theoretical Models of SI.

To address these issues, this study illustrates the theoretical landscape of SI processing and CDRM as a novel contribution to this field. The model integrates insights from Neo-Gricean pragmatics, Post-Gricean pragmatics, and Equilibrium Semantics, while introducing Diachronic-Developmental Equilibrium as a fundamental theoretical advancement. Traditional accounts primarily focus on synchronous, context-dependent inferencing. CDRM formalizes diachronic equilibrium, accounting for both real-time (synchronous) and developmental (diachronic) dynamics, particularly in child language acquisition. Unlike previous theories, CDRM accounts for: (1) cognitive load and language development balance: the model integrates how children’s limited processing resources influence implicature derivation. (2) age-sensitive pragmatic adaptation: recognizing that implicature processing undergoes a gradual refinement across developmental stages, influenced by linguistic exposure and cognitive maturation. (3) pragmatic tolerance and gradable term sensitivity: unlike adults, children exhibit a higher tolerance for underinformative statements, shaping their implicature interpretation (see Figure 1). CDRM thus offers a more comprehensive framework that not only reconciles prior pragmatic theories but also generates neurally testable predictions for SI processing.

To empirically realize this aim, the current study is designed around several key, theory-motivated innovations that are directly justified by the CDRM framework. First, it is among the first to systematically characterize the developmental neurocognitive trajectory of SI processing in Mandarin-speaking preschoolers across three consecutive age groups (4, 5, and 6 years) using ERP. This longitudinal-like design is theoretically motivated by the CDRM’s emphasis on diachronic equilibrium and is essential for capturing the gradual, resource-dependent progression hypothesized for Mandarin children, whose acquisition is uniquely shaped by linguistic-specific factors such as quantifier ambiguity and lower input frequency. Second, it employs an ecologically valid, context-embedded paradigm using animated scenarios. This methodological choice is explicitly motivated by the model’s assumption that SI derivation in Mandarin is highly context-dependent, and allows for the examination of meaning negotiation in settings that approximate natural interaction—a significant advancement over decontextualized judgment tasks commonly used in earlier studies. Third, it directly proposes and tests the CDRM as a novel framework that integrates diachronic equilibrium with resource-constrained processing. The model generates specific, testable predictions regarding the attentional (P200), semantic (N400), and pragmatic (LPC) dynamics of SI derivation across development. Together, these design features enable the study to address how Mandarin-speaking children acquire pragmatic competence under distinct linguistic and cognitive constraints, thereby bridging language acquisition, cognitive science, and developmental neuroscience through a unified theoretical model. By bridging language acquisition, cognitive constraints, and neural adaptation, CDRM marks a paradigm shift in understanding pragmatic development in children and provides a principled foundation for the present investigation. Thus, this study addresses the following three key questions. These research questions are theory-driven rather than purely descriptive. In particular, Questions 2 and 3 are designed to evaluate model-level predictions derived from the Cognitive-Dynamic Relevance Model (CDRM). They are empirically operationalized through specific experimental manipulations (contextual felicity), age-group comparisons, and ERP component analyses (P200, N400, LPC), which together allow us to examine how multidimensional cognitive constraints interact in real time and how these dynamics inform theoretical refinement. Framing the questions at both the developmental and model-evaluative levels enables the study to link neural evidence with theoretical accounts of pragmatic inference:

1. What are the developmental characteristics exhibited by Chinese preschoolers aged 4 to 6 when processing SI?
2. How does the interpretation of SI by Chinese preschoolers emerge from the real-time interaction of multidimensional constraints as conceptualized in the Cognitive-Dynamic Relevance Model (CDRM)?
3. How do the observed developmental ERP patterns inform, extend, or revise existing theoretical models of SI processing from a neurocognitive–pragmatic perspective?

Based on prior developmental, ERP, and cross-linguistic findings on scalar implicature, as well as the resource-constrained assumptions of the CDRM framework, we formulate the following predictions:

(1) Early attentional processing (P200) will show age-dependent contextual sensitivity. Specifically, younger preschoolers (especially 4-year-olds) are expected to exhibit stronger P200 modulation across contextual conditions, reflecting greater attentional allocation and less efficient attentional gating under infelicitous contexts.
(2) Semantic integration processing (N400) is predicted to be sensitive to contextual felicity across age groups, with infelicitous contexts eliciting larger N400 amplitudes than felicitous contexts, indicating increased semantic integration demands. However, given prior mixed developmental findings, age differences in N400 magnitude are treated as an open empirical question rather than a directional prediction.
(3) Late-stage pragmatic-memory processing (LPC/P600-range positivity) is expected to show context-related modulation associated with pragmatic reevaluation and memory updating. In line with developmental constraint accounts, LPC effects may emerge across age groups but are not assumed to show a strong age-gradient increase within the 4–6-year range.

Together, these predictions test the CDRM claim that scalar implicature processing in preschoolers is characterized by component-specific developmental sensitivity, with early attentional stages showing stronger age effects and later semantic–pragmatic stages showing more stable context-driven effects.

## 3. Research Design and Experimental Procedure

### 3.1. Experimental Participants

Based on previous’ findings (Wilson et al., 2020; Papafragou & Tantalou, 2004; Teresa Guasti et al., 2005), this study recruited 54 native 4 to 6 years old Chinese preschoolers as research participants and divided them into three groups: lower preschoolers (4 years old, n = 18), middle preschoolers (5 years old, n = 18), and higher preschoolers (6 years old, n = 18). All participants were typically developing (neurotypical) children, with no reported history of neurological, developmental, or psychiatric disorders, as confirmed by parental report at recruitment. All children came from middle-income families, with parents holding at least a college degree and children with speech or hearing impairments were excluded. During preparation, researchers obtained “Parental Informed Consent Form” from the parents of all participating children.

Before the formal experiment, a pilot study was conducted with two children from each age group to assess the feasibility and effectiveness of the experimental design. A total of 54 children initially participated in the formal experiment. However, due to unsuccessful EEG data collection (n = 3) or personal reasons (n = 2), the final dataset included valid data from 49 participants: 15 from the lower preschoolers group, 17 from the middle preschoolers group, and 17 from the higher preschoolers group. An a priori power analysis (α = 0.05, power = 0.80, effect size f = 0.25) recommended a minimum sample of 42 participants. Our final sample (N = 49) exceeded this threshold.

### 3.2. Experimental Materials

Following the conventional semantic–pragmatic distinction in SI processing, we operationalized two contextual conditions. In a felicitous context, the quantifier “some” is interpreted in accordance with its lower-bound semantic meaning, that is, it is satisfied whenever at least part of the relevant set holds, including cases where the whole set holds (“at least some, possibly all”). For instance, a statement like “Some guests have arrived” would be compatible with a scenario in which all guests have arrived. Conversely, in an infelicitous context, the same utterance becomes pragmatically under-informative if all is true, and therefore more strongly biases an upper-bound pragmatic interpretation (“some but not all”). Therefore, the listener infers that not all guests have arrived, based on contextual cues and expectations of informativeness. To address the issue of validation for the “felicitous/infelicitous” context operationalization, we included validation data from adult controls (n = 20). These adults confirmed that infelicitous contexts reliably elicit pragmatic interpretations of “yixie” (i.e., “at least some, but not all”). Additionally, inter-rater agreement for context classification was reported, with Krippendorff’s α = 0.85, confirming strong consistency in context categorization.

In order to simulate real-life interactive scenarios with natural hierarchical implications, we designed dynamic visual stimuli. The animations presented common dialogical situations involving “some” (with the implicitly replaceable stronger term “all”). To control for potential interference related to visual salience, we systematically balanced the animation complexity by standardizing motion trajectories, object sizes, and color contrasts. All animations retained only necessary background elements to reduce additional cognitive load. Prior to the formal experiment, we randomly selected 20% of the animated scenes and required children to answer logical consistency judgment questions based on the scenes (e.g., “Are all/some of the cabbages under the elephant’s feet?”). Only materials with an accuracy rate of ≥90% were retained, ultimately excluding eight scenes and preserving 140 for the main study.

To ensure the validity and appropriateness of the experimental materials, a systematic piloting and validation procedure was implemented, adhering to the following criteria and sequence.

First, concerning the perceptual quality of the animations themselves, twenty native Mandarin-speaking adults independently rated the naturalness and clarity of the animations on 7-point Likert scales. Inter-rater reliability was excellent (Krippendorff’s α = 0.82 for naturalness; α = 0.88 for clarity). Subsequently, a separate group of fifteen adult raters provided independent evaluations. High mean scores were obtained for both naturalness (M = 6.4, SD = 0.8) and clarity (M = 6.7, SD = 0.6). One-sample t-tests confirmed that both ratings were significantly above the scale midpoint (*p*s < 0.001), indicating that the animations effectively conveyed the intended contrasts between experimental conditions.

Second, to validate the core semantic manipulation (felicitous vs. infelicitous) for the child population, ten preschoolers who did not participate in the main study completed a forced-choice task (e.g., “Does *some* mean *not all* here?”). The agreement between children’s responses and the predefined adult classification was moderate (Krippendorff’s α = 0.72), confirming the contextual validity of the materials for the target age group.

Third, to ensure the animations effectively captured and guided children’s attention, an eye-tracking pilot test was conducted. Results showed that 90% of children fixated on the target stimuli within 500 ms post-onset, demonstrating that the materials reliably elicited visual attention to the critical scene elements, a prerequisite for online processing studies.

Finally, based on the above validation steps, the final set of animation materials was selected for the main experiment which consisted of 140 animated trial videos. Their presentation was programmed using E-Prime 3.0 software. To manage cognitive load and maintain the attention of our young participants, the trials were organized into 7 blocks, with each block containing 20 trials. This block design allowed for mandatory breaks between blocks to counteract fatigue, facilitated the randomization of trial presentation to mitigate order effects, and helped sustain children’s engagement throughout the session.

These materials were not only perceptually clear and natural, effectively distinguishing between experimental conditions, but also conveyed semantic manipulations understandable to children and reliably guided their visual attention, thereby establishing a solid foundation for subsequent EEG data acquisition. Importantly, within the CDRM framework, resource-related constraints are interpreted at a neurocognitive level and are inferred from ERP response patterns and contextual modulation effects rather than directly indexed through independent cognitive assessments (e.g., working memory or executive function tests). We note that these resource-related interpretations are inferential in nature and are derived from neural response patterns rather than independent cognitive assessments. They are intended to complement, rather than replace, broader maturation-based developmental explanations. This clarification helps distinguish ERP-based resource allocation accounts from more general age-related developmental effects. Additionally, prior to the main experiment, all children completed a brief familiarization task involving listening to AI-synthesized speech with neutral content (e.g., “look at the elephant”). This was designed to help them to get used to the characteristics of the synthesized voice and reduce potential cognitive load associated with voice novelty.

### 3.3. Experimental Process

**Figure 2:**
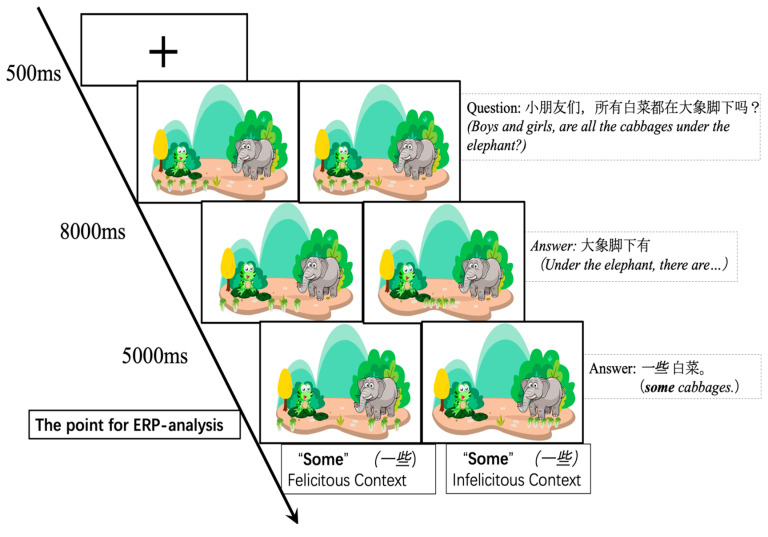
Experimental flowchart.

In the process of video production, the duration of each frame was kept equal to minimize the potential interference of duration differences on the results. At the 2 s mark, five identical objects (e.g., cabbages) appear under the frog’s feet. Subsequently, three or five objects slowly move under the elephant’s feet. At the 3.12 s mark, the first auditory stimulus is played, presenting the background sentence for 3.06 s, such as “Boys and girls, are all the cabbages under the elephant?”. Then, the animation holds a still frame for 1 s, and at the 4.06-s mark, the second auditory stimulus is played, which is the test sentence containing “some”, such as “Under the elephant, there are some cabbages”, with a playback duration of 3.08 s. A response marker is set to indicate “some”. After all sound stimuli have concluded, the video frame remains static for 0.5 s to allow participants to make judgment (see Figure 2).

### 3.4. Experimental Procedure

The experiment was conducted in a quiet room, with each child tested individually to minimize external interference and ensure the validity of the results. To minimize distraction and ensure EEG recording quality, parents were not present in the testing room during data collection. However, they remained in an adjacent waiting area and could be immediately invited into the room if the child showed discomfort or requested parental presence. Before the formal experiment, the researcher played practice videos, explaining the tasks in example (1) and children are asked to press the key “J” or “F” to judge whether the response (B) was appropriate.

Example (1): A: *Boys and girls, are all the apples under the elephant?*

B: *Under the elephant, there are some apples.*

During the experiment, children who performed well received small gifts after completing each group of videos. The total testing time for the formal experiment was approximately 25 to 30 min. To reduce fatigue and improve the data accuracy, a flexible rest mechanism was implemented: participants could take short breaks after completing each group of tests to refocus and maintain the effectiveness of the experiment.

### 3.5. Collection and Processing of EEG Data

During experiment, the children wore EEG caps and sat upright in front of the screen, keeping eyes fixated on the central stimulus and maintaining a viewing distance of 100 cm. Auditory stimuli were delivered through the laptop’s built-in speakers at an average volume of 65 dB. Visual stimuli were presented centrally on a screen measuring 21.6 × 14.4 cm. To optimize data quality, the size and position of the stimuli were carefully controlled to minimize eye movement artifacts and ensure stable foveal processing. At a fixed viewing distance of 100 cm, the stimuli subtended horizontal and vertical visual angles of approximately 10.0° and 8.2° respectively. This configuration ensured that the stimuli remained well within the central visual field, which is a standard practice in EEG studies to reduce ocular artifacts and maintain consistent visual input across participants.

EEG data were recorded using a portable ANT Neuro eego™ system (version 1.9.2) with a 64-channel waveguard™ EEG cap, adhering to the international 10–20 system. During online data collection, the reference electrode was positioned at the CPz point, and the grounding electrode was set to GND. This online referencing scheme was adopted for two primary considerations. Theoretically, placing the reference at a midline scalp location like CPz provides a relatively stable and electrically neutral signal during recording, which serves as a robust foundation for subsequent offline re-referencing to a common average or other desired configuration. Practically, for studies involving a pediatric population, a vertex or central–parietal reference offers enhanced physical stability compared to mastoid or earlobe placements, minimizing the risk of displacement due to participant movement and thus improving data integrity throughout the recording session (Da Silva et al., 2010).

The sampling mode was direct current (DC), with a bandwidth range of DC (0 Hz) to 0.26 × 1064.96 Hz. The amplifier model used was EEG64-CA-208. Additionally, electrooculography (EOG) channels were simultaneously recorded, with a sampling rate of 4096 Hz per channel. The electrode-skin contact resistance was maintained below 5 kΩ throughout the experiment. To preserve the integrity of the original signals, no pre-filtering was applied during data collection.

Matlab R2021a data processing software with the ERPlab extension package was used to analyze offline EEG data. First, all electrodes were localized, and the EOG, M1, and M2 channels were excluded, employing a whole-brain average reference. Following established guidelines for children’s EEG experiments (Männel, 2008), artifacts with frequencies exceeding 30 Hz or below 0.3 Hz were filtered out. The ERP data were segmented into a time window spanning from 200 ms before the onset of the auditory stimulus to 800 ms after its presentation, and baseline correction was applied. This time window was selected to comprehensively encompass the temporal dynamics of the cognitive processes critical for scalar implicature derivation, including early perceptual processing, semantic integration (N400), and later-stage pragmatic inference (P600/LPC). Its duration aligns with the standard practice in developmental ERP research on language processing (Luck & Kappenman, 2011). Artifact removal was conducted using ICA, with manually identifying and removing artifact signals (Onton et al., 2006). ICA components exceeding ±100 μV or showing stereotypical ocular/muscular noise were manually rejected by two blinded experts (inter-rater agreement: κ = 0.91). Afterward, the ERP data were averaged and further grouped by age category, which resulted in three grand-average EEG datasets for subsequent analysis.

### 3.6. Analysis of EEG Data

Three ERP components were selected on the basis of prior research on SI processing and pragmatic inference. ERP components of P200 (early attentional allocation), N400 (semantic integration), and LPC (late pragmatic evaluation) have been widely reported in SI-related ERP studies (Nieuwland et al., 2010; Panizza et al., 2021).

We did not include MMN (Mismatch Negativity), as our study focused on explicit pragmatic inference rather than pre-attentive auditory discrimination (Pulvermüller & Shtyrov, 2006). MMN primarily reflects early-stage automatic phonological processing and is thus less relevant to controlled inferential processes in scalar implicature derivation.

**Figure 3:**
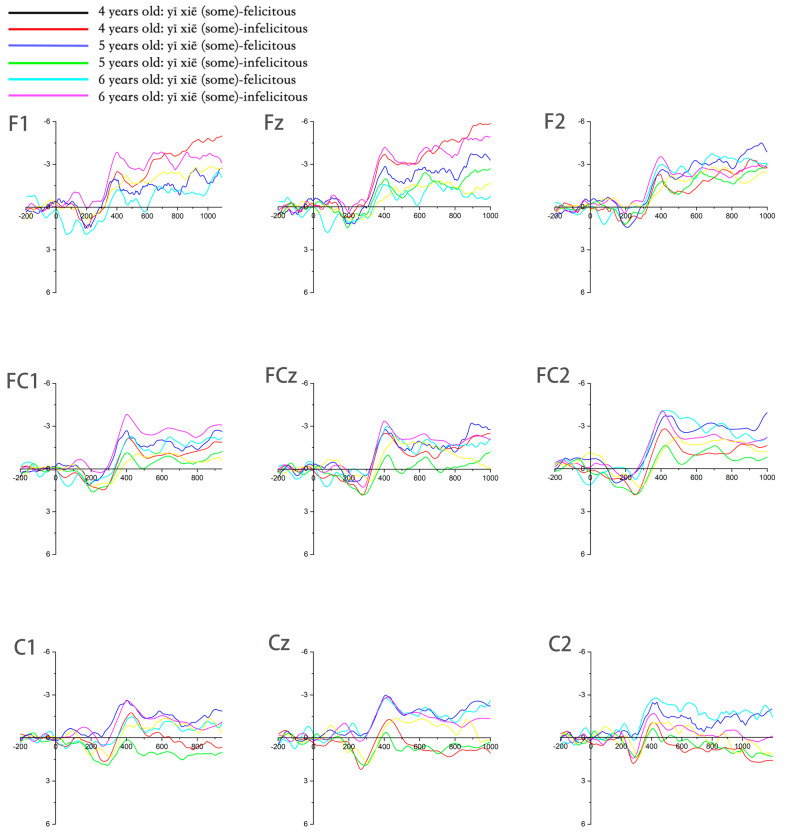
Comparative characteristics of ERP waveform based on contextual conditions.

Based on the processed ERP data, grand-average waveforms across the three age groups were plotted at four electrode sites: FCz, Cz, CPz, and Pz (see Figure 3). The data revealed that both felicitous and infelicitous contextual conditions elicited a distinct positive deflection, the P200, peaking within the 150–250 ms time window post-stimulus onset. This was followed by a negative deflection, the N400, most prominent within the 250–500 ms window and particularly evident over central–parietal sites (CPz, Pz). Subsequently, a relatively sustained Late Positive Component (LPC) emerged around 500 ms.

To systematically analyze the ERP characteristics across age groups and align with established research, mean amplitudes for the scalar term “some” (*yì xiē*) were extracted under both contextual conditions within the following time windows: P200 (150–250 ms), N400 (300–500 ms), and LPC (500–700 ms). The extracted amplitude data were then analyzed using SPSS 22.0 software. Multi-factorial repeated-measures ANOVAs with the factors Group (age), Contextual Condition (felicitous/infelicitous), and Electrode Site were conducted to explore significant differences and interaction effects for each component. In addition, the mixed-effects models reported in the Results were fitted in R (R Core Team, version 4.3.0) using lme4 (lmer) with lmerTest for *p*-value estimation (D. Bates et al., 2015).

Guided by the typical scalp topographies reported in prior literature, separate three-way repeated-measures ANOVAs were performed for each component, focusing on electrode clusters corresponding to their maximal distributions. For the P200 component (150–250 ms), which is associated with early attentional allocation and visual processing, often maximal over parieto-occipital regions (Potts et al., 2004), the analysis was conducted for the factors Group (3: lower, middle, higher preschoolers) × Contextual Condition (2: felicitous, infelicitous) × Electrode Site (4: O1, PO3, PO5, PO7). Critically, to dissociate activity related to higher-order auditory-language processing from low-level visual perception, P200 amplitudes at fronto-central sites of interest (Fz, Cz, FCz) were explicitly contrasted with those from occipital sites (O1, O2). A significant effect confined to the fronto-central region would support the interpretation that the observed P200 modulation is specific to cognitive evaluation rather than general perceptual differences (Kandel et al., 2010). For the N400 component (300–500 ms), which is known to manifest maximally over central–parietal scalp sites during semantic integration (Kutas & Federmeier, 2011), the analysis included the factors Group × Contextual Condition × Electrode Site (6: F2, F4, FC2, FC4, C2, Cz). For the LPC (500–700 ms), which is linked to late pragmatic reevaluation and memory updating and typically shows a medial-posterior maximum (Friedman & Johnson, 2000), the analysis was performed for the factors Group × Contextual Condition × Electrode Site (6: FC1, FC2, C1, C2, CP1, CP2).

Following the analysis of main effects and interactions for each variable within the specified time windows, the Bonferroni correction was applied for post hoc testing. This approach ensured the accuracy of the statistical results while addressing the issue of multiple comparisons.

## 4. Results

### 4.1. Behavioral Responses of Preschoolers to the Scalar Alternative “Some”

**Figure 4:**
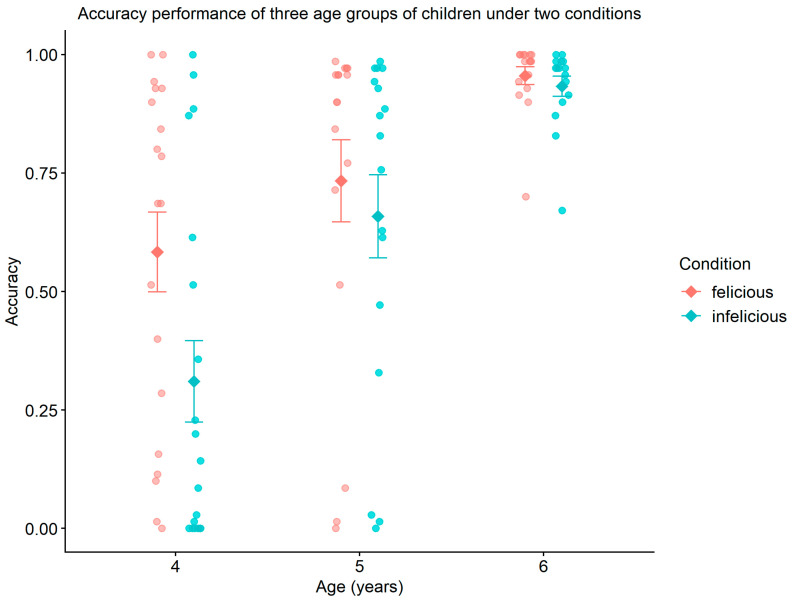
Accuracy performance of three age groups of children under two conditions.

The mixed two-factor analysis of variance (ANOVA) for different age groups of children under two conditions regarding the semantic and pragmatic interpretation of “some” revealed a significant main effect of age ($F\left(2,94\right)=26.687,p=0.000\;(p<0.001),{\eta \rho }^{2}=0.362)$. This indicates that children’s understanding of the semantic and pragmatic meanings of “some” improves significantly as they age. The main effect of contextual conditions was not significant, indicating no significant difference in accuracy between the felicitous and infelicitous contextual conditions $\left(F\left(1,94\right)=26.687,p=0.057,{\eta \rho }^{2}=0.038\right)$. Furthermore, the interaction between age and contextual conditions was also not significant, suggesting that the impact of contextual conditions on accuracy does not vary significantly across different age groups ($F\left(2,94\right)=1.300,p=0.277,{\eta \rho }^{2}=0.027)$. The statistical results are presented in Figure 4.

An ANCOVA was conducted with animation comprehension accuracy as a covariate, and the results showed that comprehension did not significantly moderate accuracy (*F*(1,47) = 1.32, *p* = 0.257), indicating that the effectiveness of the context manipulation was not influenced by individual differences in comprehension.

**Figure 5:**
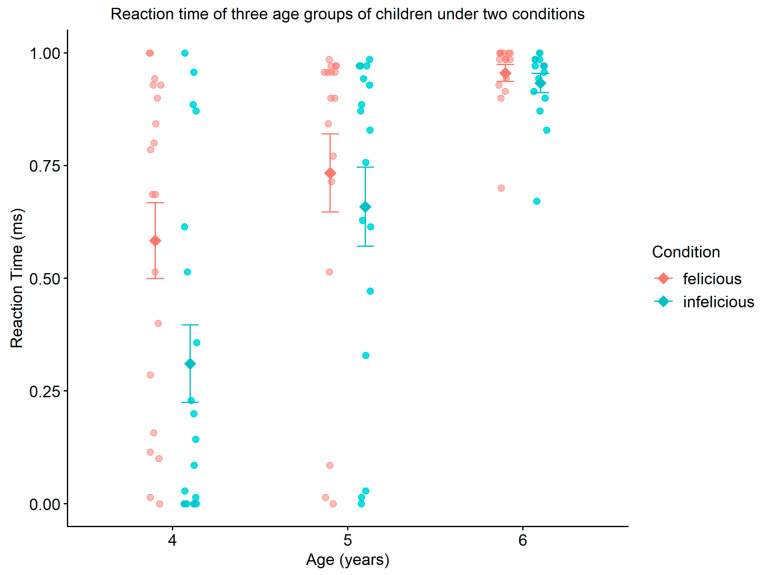
Reaction time of three age groups of children under two conditions.

Furthermore, regarding the reaction times of children in different age groups under two conditions for “some”, the mixed two-factor analysis of variance (ANOVA) revealed a significant main effect of age $\left(F\left(2,98\right)=8.402,p=0.000\;(p<0.001),{\eta \rho }^{2}=0.146\right)$. This indicates that children’s cognitive processing speed for “some” improves notably with increasing age. The main effect of contextual conditions was not significant, suggesting no significant difference in reaction time between the felicitous and infelicitous conditions $\left(F\left(1,98\right)=0.013,p=0.908,\;{\eta \rho }^{2}=0.000\right)$. Additionally, the interaction between age and contextual conditions was not significant $\left(F\left(2,98\right)=0.205,p=0.815,\;{\eta \rho }^{2}=0.004\right)$, indicating that the influence of contextual conditions on reaction time does not differ significantly across age groups (see Figure 5).

### 4.2. Preschoolers’ Cognitive Neural Processing of the Scalar Alternative “Some”

**Figure 6:**
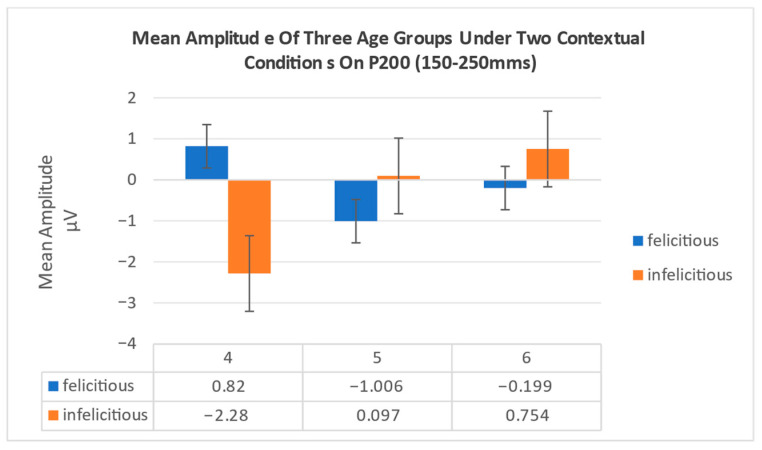
Mean amplitude of three age groups under two contextual conditions on P200.

In P200 (150–250 ms), the results showed that the main effect of contextual condition was not significant$,\;F\left(1,45\right)=0.311,p=0.580,{\eta \rho }^{2}=0.007;$ the main effect of group was also not significant, $F\left(2,45\right)=1.333,p=0.274,{\eta \rho }^{2}=0.056$; but the interaction effect between group and contextual condition was significant$,\;F\left(2,45\right)=4.577,p=0.016,{\eta \rho }^{2}=0.169$ [0.06, 0.32]. Simple effect analysis results show that the lower preschoolers exhibits significant differences between felicitous and infelicitous conditions, $F\left(1,45\right)=7.278,p=0.010,{\eta \rho }^{2}=0.139$ [0.03, 0.29], with a significantly greater P200 amplitude in the felicitous condition. In contrast, middle and higher preschoolers showed no significant differences in P200 amplitude between the two conditions (*p* > 0.05). Under infelicitous conditions, lower preschoolers exhibited significantly greater P200 amplitudes compared to higher preschoolers, $F\left(2,45\right)=4.980,p=0.011,{\eta \rho }^{2}=0.181$ [0.07, 0.34]. Additionally, a marginal difference was observed between lower and middle preschoolers (*p* = 0.065), with lower preschoolers showing noticeably greater amplitudes (see Figure 6).

To rule out the potential confound of basic perceptual comprehension on the P200 effect, an Analysis of Covariance (ANCOVA) was conducted with animation comprehension accuracy as a covariate. The results showed that comprehension accuracy did not significantly predict P200 amplitude (β = 0.11, SE = 0.09, t = 1.22, *p* = 0.224), supporting the notion that the P200 differences stem from contextual conditions rather than perceptual features.

**Figure 7:**
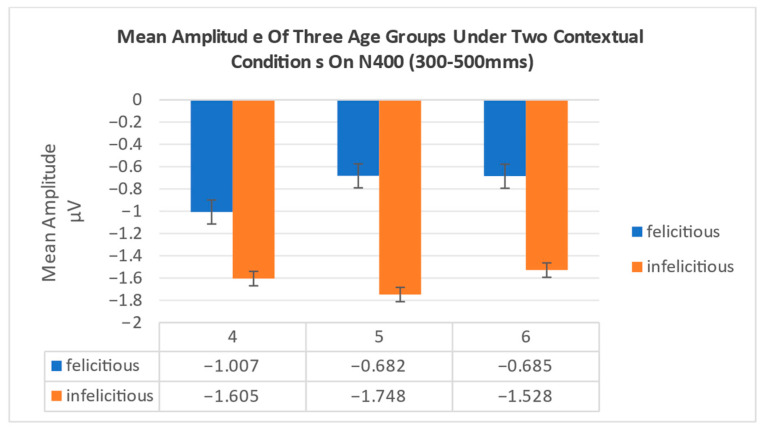
Mean amplitude of three age groups under two contextual conditions on N400.

In N400 (300–500 ms), the results showed significant main effects of contextual condition, $F\left(1,45\right)=4.196,p=0.046,{\eta \rho }^{2}=0.085\left[0.01,0.22\right]$, with all participants exhibiting a greater N400 under infelicitous condition (Μ=−1.627 μV) compared to felicitous condition $\left(Μ=-0.791\;\mu V\right).$ The main effect of group was not significant, $F\left(2,45\right)=0.028,p=0.972,{\eta \rho }^{2}=0.001$ and the interaction effect between contextual conditions and group was not significant, $F\left(2,45\right)=0.106,p=0.899,{\eta \rho }^{2}=0.005$ (see Figure 7).

**Figure 8:**
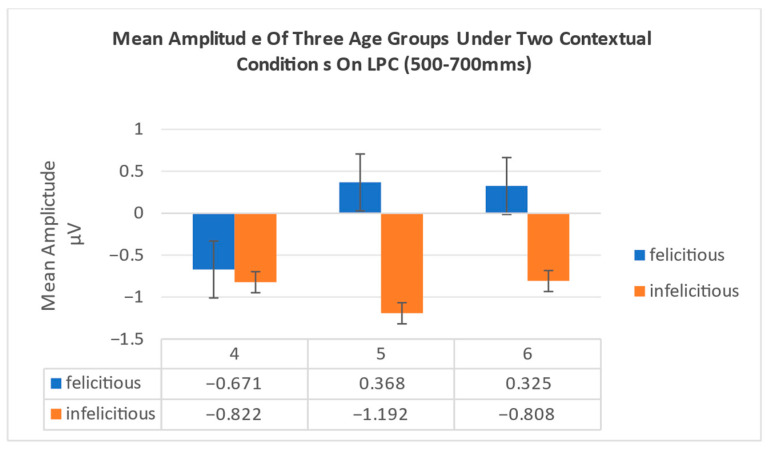
Mean amplitude of three age groups under two contextual conditions on LPC.

In LPC (500–700 ms), the results showed that the main effect of the two conditions is marginally significant, $F\left(1,45\right)=2.956,p=0.092,{\eta \rho }^{2}=0.062[0.00,0.18]$, and all participants under infelicitous contextual condition (Μ=−0.941 μV) elicited greater LPC than felicitous condition (Μ=0.007 μV). The main effect of the age group is not significant, $F\left(2,45\right)=0.123,p=0.884,{\eta \rho }^{2}=0.005$. The interaction effect between the contextual conditions and the age group is not significant, $F\left(2,45\right)=0.543,p=0.585,{\eta \rho }^{2}=0.024$. The interaction effect among the contextual conditions, age group, and electrode is not significant $\left(F\left(10,225\right)=1.117,p=0.336,{\eta \rho }^{2}=0.047\right)$ (see Figure 8; Table 1).

To examine the relationship between neural responses and behavioral performance, we fitted a mixed-effects model predicting accuracy from N400 mean amplitude, with random intercepts for subjects and items (Accuracy ~ N400 amplitude + (1|Subject) + (1|Item)) in R (lme4; lmerTest) (D. Bates et al., 2015). The results revealed a significant positive association between N400 amplitude and accuracy (*p* < 0.01), indicating that trials eliciting larger N400 responses were more likely to be correctly interpreted. This relationship remained significant when age group and contextual condition were included as additional fixed effects, suggesting that the observed association was not driven solely by developmental stage or task condition.

Importantly, parallel models including P200 or LPC amplitudes as predictors did not yield significant effects (*p*s > 0.10), indicating that the brain-behavior relationship was specific to the N400 time window rather than reflecting a general electrophysiological sensitivity. These findings suggest that individual variability in semantic integration processes, as indexed by the N400 component, plays a critical role in successful scalar implicature comprehension.

Taken together, the behavioral and ERP results reveal a developmentally specific and component-selective pattern of scalar implicature processing in preschool children. Behaviorally, performance improved with age, while contextual manipulation did not yield uniform accuracy or reaction time differences across groups. At the neural level, early attentional processing (P200) showed age-dependent contextual sensitivity limited to younger preschoolers, whereas semantic integration processes (N400) exhibited a robust effect of contextual felicity across age groups. In contrast, late positive activity (LPC) showed only a marginal sensitivity to contextual conditions and did not display stable age-related modulation.

Crucially, the observed ERP effects were not attributable to differences in perceptual comprehension, as confirmed by covariate analyses, nor were they driven by general task difficulty. The selective association between N400 amplitude and behavioral accuracy further underscores the functional relevance of semantic integration processes in scalar implicature interpretation. These findings provide converging evidence that scalar implicature development during early childhood is characterized by stage-specific neural signatures rather than a uniform maturational trajectory, setting the stage for the theoretical interpretation presented in the following Section 5.

## 5. Discussion

This study employed ERPs to investigate SI processing in Chinese preschoolers under felicitous and infelicitous contextual conditions, with a focus on developmental differences in neurocognitive processing mechanisms rather than behavioral outcomes alone. Neural evidence revealed age-related variation in attention allocation (P200), semantic integration (N400), and late-stage pragmatic and memory-related processing (LPC), while behavioral accuracy primarily showed age effects without strong contextual modulation.

We begin by explicitly revisiting our three original research questions and hypotheses. First, regarding the developmental trajectory of SI processing (RQ1), both ERP and behavioral results indicate a graded developmental progression: 4-year-olds showed heightened P200 responses under infelicitous conditions, consistent with attentional overload, whereas 5- and 6-year-olds demonstrated more efficient neural resource allocation and more stable interpretive patterns. Second, concerning the underlying processing mechanism (RQ2), the findings support the Cognitive-Dynamic Relevance Model (CDRM): SI derivation emerges from dynamic interaction among attentional control (P200), semantic integration (N400), and late pragmatic-memory integration processes (LPC), each indexing a distinct processing stage. Third, with respect to theoretical expectations and cross-linguistic implications (RQ3), the results indicate that SI computation remains effortful and resource-dependent across early childhood, thereby challenging strong default accounts, and further suggest that Mandarin-specific factors (e.g., quantifier ambiguity and lower input frequency) may modulate the developmental timeline at the neural level.

Together, these results provide a structured empirical answer to our initial questions and set the stage for the more detailed theoretical interpretation developed in the following sections.

We note that interpretations concerning resource constraints within the CDRM framework are inferential and are based on ERP component dynamics (P200, N400, and LPC) and their contextual modulation patterns, rather than on independent cognitive or executive-function measures. These components are treated as neurocognitive proxies of attentional allocation, semantic integration load, and late-stage inferential control. Accordingly, resource-allocation accounts proposed here are intended to complement rather than replace broader maturation-based developmental explanations. This clarification delineates ERP-based processing interpretations from general age-related developmental capacity.

### 5.1. Developmental Characteristics of Preschoolers’ Processing of SI

The results demonstrate clear developmental characteristics in SI processing among children aged 4 to 6. Specifically, during early stages, significant age differences were observed in the P200, while N400 and LPC did not exhibit significant age-related differences in the later stages.

#### 5.1.1. Developmental Characteristics in the Early Stage (150–250 ms)

The P200 component is an early ERPs marker associated with semantic processing, reflecting the semantic relevance of non-literal meaning expressions preceding the context (Federmeier et al., 2005). P200 has been linked to the visual search, attentional allocation, language context integration, memory, and repetition effects (Freunberger et al., 2007). The P200 effect in 4-year-olds may reflect immature attentional control (Hillyard & Münte, 1984), though source-level analysis is needed to confirm prefrontal engagement. In the context of SI, S. Zhao et al. (2021) discovered that pragmatic processing of “some” induces a significant P200 effect in the frontal lobe. This over-allocation of cognitive resources reflects the immature attentional gating mechanism in younger children, with older children showing more efficient resource allocation due to top-down contextual predictions.

Combining prior findings, this study highlights that P200 in lower preschoolers under infelicitous was significantly greater than that of older preschoolers. This suggests that in infelicitous condition, the inconsistency between the semantic content of the visual stimulus and the contextual information makes lower preschoolers more susceptible to external visual stimuli, which leads to an over-allocation of attention resources. Consequently, processing pragmatic meaning implied by “some” is challenging for lower preschoolers. In contrast, older preschoolers exhibit greater ease in pragmatic processing within infelicitous contexts, as they are less influenced by external information and require fewer attention resources for successful processing. The age-related differences in P200 may reflect the maturation of the attentional gating mechanism: four-year-old children need to suppress irrelevant visual cues (e.g., interference from redundant objects in the animation) in infelicitous contexts, leading to excessive mobilization of prefrontal attentional resources. In contrast, six-year-old children optimize attentional allocation efficiency through top-down contextual prediction.

Meanwhile, lower preschoolers exhibit significantly greater P200 in felicitous than infelicitous condition, suggesting that lower preschoolers tends to focus more on literal meaning of the alternative, while paying less attention to pragmatic meaning when constrained by different contexts. By behavioral results, lower preschoolers are more inclined to interpret “some” as “all”, which interprets semantic meaning is more readily acquired than pragmatic meaning during the early stages of cognitive processing.

On the other hand, there is no significant difference on P200 for middle and older preschoolers in both contexts, which suggests that both groups can draw upon their existing cognitive experiences and situational context to effectively allocate attentional resources, demonstrating a tendency towards consistency in their cognitive strategy application. However, a closer analysis of the behavioral results reveals that older preschoolers are more inclined to interpret “some” in infelicitous contexts as “at least some but not all” (i.e., pragmatic interpretation). In contrast, middle preschoolers tend to adopt a dual interpretation model, incorporating both semantic and pragmatic meanings for “some”. Moreover, older preschoolers exhibit significantly faster response time in both contexts compared to middle preschoolers. These findings suggest that middle preschoolers (5 years old) are at a developmental trajectory driven by cumulative linguistic, cognitive, and contextual factors for processing SI, where their ability to infer pragmatic meanings remains relatively unstable.

Overall, middle preschoolers are at a developmental trajectory for processing SI with instable pragmatic ability, while older preschoolers demonstrate a significant advancement in processing SI. This finding highlights the age-specific developmental trajectory in children’s transition from relying on semantic meaning to comprehending pragmatic meaning during SI processing.

#### 5.1.2. Developmental Characteristics in the Mid Stage (300–500 ms) and Late Stage (500–700 ms)

During the mid and late processing stages, no significant differences were observed under two conditions among three age groups. However, the amplitudes of N400 and LPC across all age groups under the infelicitous condition were significantly greater than those under felicitous one. Several factors may account for this finding. Rather than representing separate effects, the N400 and LPC differences under infelicitous conditions are better interpreted as a temporally ordered processing sequence. At the mid processing stage (300–500 ms), corresponding to the N400 window, infelicitous contexts increase semantic integration demands through two closely related mechanisms: increased semantic mismatch and reduced contextual predictability.

First, semantic mismatches between the target stimulus and contextual information under infelicitous conditions require additional semantic integration and readjustment, thereby activating stronger semantic processing mechanisms and resulting in greater N400 amplitudes.

Second, contextual predictability is lower in infelicitous contexts. While felicitous contexts allow children to effectively use contextual cues to anticipate upcoming information and reduce semantic processing load, infelicitous contexts generate prediction errors and weaken contextual support, further amplifying N400 responses.

These two factors jointly characterize the semantic integration stage and should be understood as complementary mechanisms within the N400 time window rather than independent effects.

Third, at the later processing stage reflected by the LPC (500–700 ms), the processing focus shifts from semantic integration to pragmatic reevaluation and inferential control. Unlike the earlier N400 effects driven by semantic mismatch and contextual predictability, LPC effects are typically associated with controlled inferential adjustment, situational reevaluation, and working memory-supported integration. LPC is widely regarded as a key electrophysiological indicator of pragmatic reasoning and late-stage inferential processing, and has been linked to situational monitoring, evaluation, response selection, and working memory engagement (Van Petten et al., 1997; Bambini et al., 2016). The LPC consistency across the three age groups therefore suggests convergence in late-stage inferential adjustment mechanisms rather than a repetition of earlier semantic processing load.

Lastly, developmental differences in pragmatic and semantic processing abilities among three age groups could also contribute to these findings. All preschoolers exhibited greater N400 and LPC amplitudes in response to infelicitous contexts, which may be because the core mechanisms of language processing, such as the activation of the semantic memory and perceptual system, remaining consistent throughout development but are still influenced by contextual factors. While prior corpus studies (Wu, 2023) suggest lower SI frequency in Mandarin child-directed speech, our study did not measure input exposure. Future work should link parental SI usage to children’s ERP profiles to confirm input-driven delays.

Compared to the findings’ of Panizza et al. (2021), results discrepancy may be attributed to differences in linguistic and cultural backgrounds. German and English cultures prioritize logical reasoning, exposing children to pragmatic reasoning practices at an earlier age, while the interpretation of “yixie” in Mandarin requires children to integrate multiple factors, such as context, cultural norms, and conversational habits, thereby increasing the inferential load. The lower frequency and higher semantic ambiguity of “yixie” in Mandarin may delay children’s acquisition of pragmatic meaning, indicating that 4-year-old children delays in pragmatic inference, with age 5 serving as a developmental trajectory period for the development of pragmatic reasoning and age 6 marking a rapid acceleration in mastery of such skills. This highlights the significant influence of linguistic and cultural environments on pragmatic development.

In summary, children’s cognitive development is a crucial foundation for SI processing, offering valuable empirical evidence for understanding the neural mechanisms underlying children’s language development.

#### 5.1.3. Implicit Pragmatic Processing and the ‘Hidden Competence’ Hypothesis

Divergent behavioral and neural patterns suggest LPC compensates for overt response errors in younger children, masking contextual effects in accuracy measures. While 4-year-olds showed heightened N400 and LPC under infelicitous conditions, their behavioral accuracy remained unchanged, indicating sensitivity to pragmatic violations despite explicit task difficulties.

This aligns with pragmatic tolerance hypothesis (Katsos & Bishop, 2011), which posits that early-stage SI processing relies on implicit mechanisms not captured by behavioral data. Thus, neural markers reveal emerging pragmatic sensitivity before explicit SI derivation stabilizes, highlighting the role of cognitive maturation and linguistic exposure in bridging this gap.

### 5.2. Cognitive Processing and Meaning Interpretation of SI in Chinese Preschoolers

**Figure 9:**
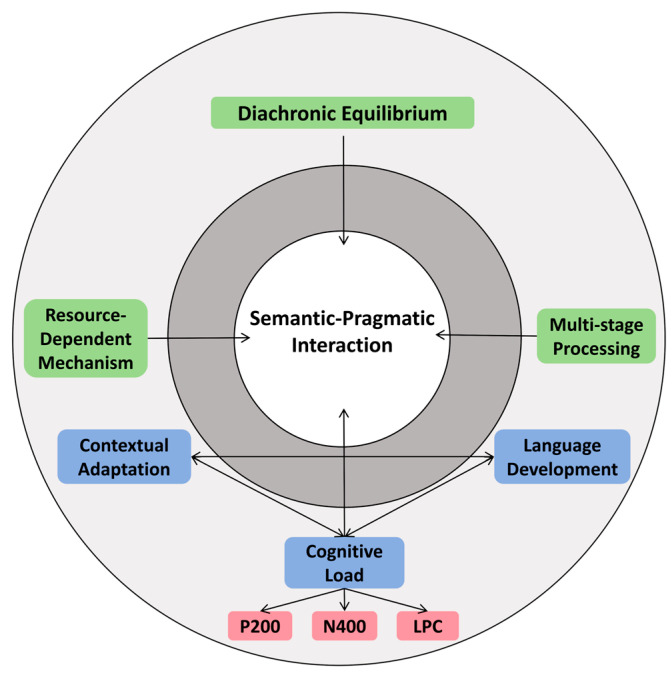
The cognitive-dynamic relevance model (CDRM) for Chinese preschoolers’ SI processing.

By integrating Neo-Gricean and Relevance frameworks, the study constructs a cognitive-dynamic relevance model for Chinese preschoolers’ SI processing (see Figure 9). The CDRM offers a system-dynamic framework for understanding scalar SI development in children, integrating semantic–pragmatic interaction, cognitive constraints, and neural evidence into a unified model.

At its core, CDRM posits that meaning interpretation is a continuous negotiation between semantic content and pragmatic inference, dynamically shaped by contextual adaptation, cognitive load, and language development. Contextual adaptation reflects the role of situational and communicative cues, while language development captures the influence of lexical and syntactic growth on children’s inferential abilities. Cognitive load, a central component of the model, is decomposed into P200 (early attentional allocation), N400 (semantic integration), and LPC (late-stage inferential processing and memory retrieval), directly linked to ERP-based neural evidence of SI processing.

Surrounding these core processes, CDRM incorporates diachronic equilibrium, emphasizing the gradual optimization of SI processing across development, and resource-dependent mechanisms, highlighting the role of limited cognitive capacity in meaning derivation. The multi-stage processing principle further underscores the sequential nature of SI computation, aligning with neurocognitive findings.

By explicitly linking cognitive load dynamics to its neural correlates (P200, N400, LPC), CDRM provides an empirically grounded, theoretically robust framework that bridges developmental linguistics, cognitive neuroscience, and pragmatic theory, offering a comprehensive model for understanding how children acquire SI over time.

The process of meaning selection is inherently dynamic, evolving over time in tandem with the development of language proficiency (Parikh, 2010). Consequently, the attribution of linguistic meaning is not static but rather a fluid balancing act shaped by diverse influences in the communicative context. Children’s language understanding is a filtering mechanism shaped by their linguistic and cognitive capacities (Li, 2004). Preschoolers, being in a critical period of language acquisition, rely on auditory signals within context to rapidly retrieve semantic information from memory and establish connections with the communicative context. This enables them to infer the implied meanings of utterances and the speaker’s communicative intentions. SI, as conceptualized by Horn (1984), exemplifies conversational implicature, encompassing both semantic and pragmatic dimensions. For preschoolers, deriving the intended meaning of scalar alternatives requires the integration of cognitive resources, communicative context and linguistic ability.

Based on prior findings, this study identifies a developmental lag in the pragmatic meaning inference of the scalar alternative “some” among younger preschoolers, suggesting that preschoolers’ interpretation of SI is characterized by instability and dynamism, which highlights the stage-specific and equilibrium-driven processes underlying their evolving ability to balance semantic and pragmatic inferences during this developmental trajectory period. The CDRM predicts that in languages with lower semantic transparency (such as Chinese), SI acquisition will be significantly later than in languages with higher semantic transparency (such as German). Our results highlight Mandarin’s delayed pragmatic integration by comparing N400 effects elicited by pragmatic violations between 300 and 500 ms-later than German’s 200–250 ms (Panizza & Onea, 2014).

In CDRM, balance emerges as the overall outcome of interactions among various internal factors within the system, which are constantly in flux. For preschoolers, language meaning selection is jointly constrained by multiple influences, making the interpretation of utterance meaning a dynamic and balanced process shaped by the interlocutor’s negotiation of these factors. Whether the interpretation leans toward semantic or pragmatic meaning, individuals dynamically integrate diverse information to select optimal meaning. Thus, the three key factors in CRDM work together to shape a state of balance, allowing for a nuanced and evolving process of meaning selection and interpretation during language development.

Within the constraints of language, children’s processing of “some” is internally influenced by their language development. Preschoolers’ language abilities and vocabulary sizes significantly increase with age (Liang, 2019). Behavioral data reveal that children between 4 and 6 years demonstrate noticeable improvements in understanding the semantic and pragmatic meanings of “some” with increasing age. Cognitive neuro data further show that lower preschoolers’ processing of “some” exhibits multi-stage characteristics, largely due to their relatively limited lexical knowledge at this developmental stage, which aligns with the “alternative-based approach” (Barner & Bachrach, 2010). Conversely, middle and older preschoolers, despite slight differences in vocabulary development, demonstrate the ability to dynamically regulate attention resources by integrating contextual information in real time. This enables them to achieve a temporary equilibrium in SI comprehension within the early processing phase. In summary, the interplay between lexical development and attention regulation within the language and cognitive systems jointly constrains children’s meaning interpretation models. These findings highlight the critical roles of both internal linguistic factors and additional cognitive resources in shaping the developmental trajectory of SI understanding.

Within cognitive constraints, children’s processing of the scalar alternative “some” is governed by the interplay of various cognitive resources. Meaning processing reflects dynamic equilibria among attention, perception, and memory. Behavioral data reveal significant age-related differences in children’s meaning selection, but cognitive neuro data indicate that preschoolers dynamically integrate contextual cues to regulate lexical semantic perception and semantic memory. At early stages, lower preschoolers’ performance is primarily regulated by lexical knowledge and attention resources, making it challenging for them to interpret its pragmatic meaning in infelicitous contexts. However, as situational context stimulates their cognitive system, their semantic perception and implicit semantic memory are activated. This dual regulation enables them to transition between semantic and pragmatic meanings of “some” across contexts. Middle and older preschoolers, while displaying similar N400 and LPC during early and late processing stages, still rely on multidimensional cognitive factors for SI comprehension. Attentional allocation, semantic activation, and memory integration jointly contribute to SI processing within a resource-constrained system. Rather than operating independently, these components function in a coordinated and compensatory manner: when one process is less efficient, others may increase their contribution to maintain overall interpretive performance. This coordinated adjustment reflects a dynamically regulated processing system shaped by cognitive load, contextual support, and developmental capacity. Therefore, preschoolers’ SI processing can be characterized as a multi-component, adaptively coordinated cognitive process in which attentional, semantic, and memory-related mechanisms jointly support meaning interpretation and pragmatic inference. Under contextual constraints, children’s understanding of “some” is influenced by the interplay of language, cognitive, and situational contexts. Linguistic context provides the foundational language ability necessary for meaning inference; situational context offers a clear communicative environment and socio-cultural background; and cognitive context supplies common knowledge, reasoning cues, ambiguity resolution, and communicative intent for interpreting meaning. Behavioral results suggest no significant difference between two contextual conditions, while ERP results show all participants elicit greater N400 and LPC under infelicitous condition. Divergent behavioral and neural patterns suggest younger children rely on implicit semantic compensation (LPC) to mask overt response error. Visual stimuli enrich the situational contexts, enabling children to activate contextually salient knowledge related to scalar alternatives. In this process, children engage in a cognitive confrontation with situational contexts, selecting the most relevant contextual hypothesis to derive SI. Lower preschoolers activated implicit semantic memory under the interactive influence of the two situational contexts, which facilitated gradual integration of cognitive contexts to derive pragmatic meaning during middle and late stages. Even behavioral results indicate middle preschoolers still possess weaker language abilities related to the knowledge of scalar alternatives compared to higher preschoolers, they effectively associated the most prominent alternative knowledge of “some” with the cognitive context. Overall, these sub-contextual factors exist in a fluid state, allowing interlocutors to integrate their recognition of implicit information within the communicative content and generate individualized interpretations of meaning.

**Figure 10:**
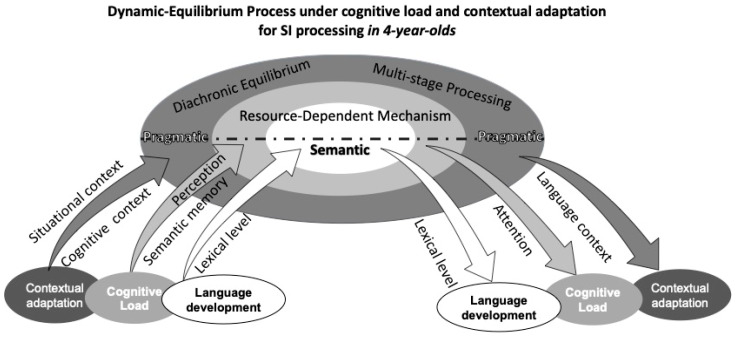
Dynamic-equilibrium process under cognitive load and contextual adaptation for SI processing in 4-year-olds.

**Figure 11:**
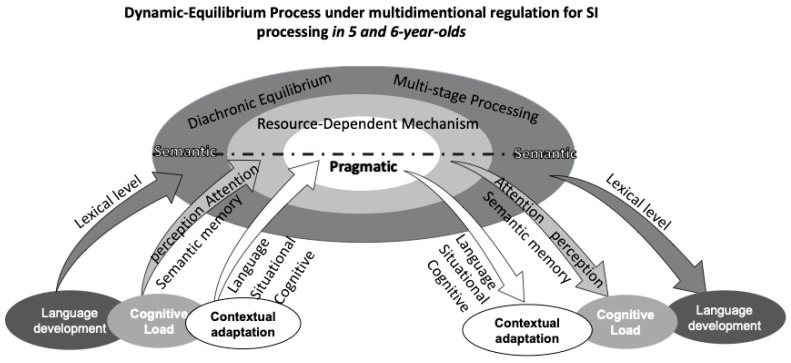
Dynamic-equilibrium process under multidimensional regulation for SI processing in 5 and 6-year-olds.

Within this framework, interactions occur between groups of constraints—language ability, cognitive development, and contextual adaptation—and the relevant influencing factors. These interactions dynamically adjust and adapt SI processing, ultimately reaching a state of equilibrium (see Figure 10 and Figure 11).

**Figure 12:**
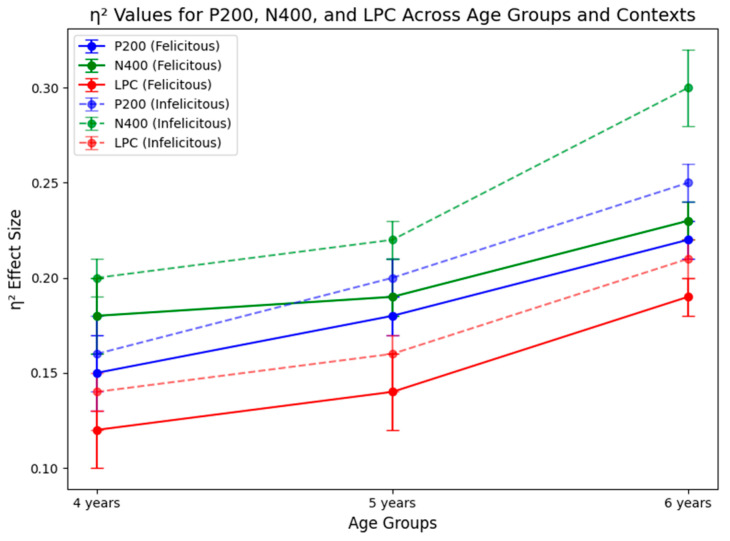
η^2^ Values for P200, N400, and LPC Across Age Groups and Contexts.

Figure 12 illustrates the developmental trajectory of effect sizes (η^2^) for SI processing across age groups, focusing on attentional allocation (P200), semantic integration (N400), and inferential reasoning (LPC) under felicitous and infelicitous contexts. Infelicitous contexts elicited stronger P200/N400 effects, with LPC showing marked growth from ages 5 to 6, aligning with CDRM’s diachronic equilibrium hypothesis. Solid lines represent felicitous conditions, while dashed lines indicate infelicitous conditions, with error bars reflecting 95% confidence intervals.

P200 effect sizes exhibit a steady increase with age, indicating a progressive refinement of attentional control. Notably, infelicitous contexts elicit consistently higher P200 effects than felicitous ones, suggesting greater cognitive effort in managing scalar implicature violations. N400 effect sizes also increase across development, with infelicitous contexts generating stronger effects, reinforcing the role of semantic integration in resolving implicature conflicts. LPC shows the most pronounced growth, particularly from 5 to 6 years, reflecting a shift toward more effortful inferential reasoning.

These findings align with the diachronic dynamic equilibrium hypothesis in CDRM, highlighting the adaptive recalibration of cognitive resources in SI processing. The increased effect sizes across all components underscore the progressive maturation of pragmatic competence, supporting a resource-dependent trajectory in implicature derivation.

### 5.3. Integrative Neurocognitive-Pragmatic Frameworks in CDRM: Toward a Multidimensional Mode

The development of SI has been extensively studied within Neo-Gricean, Post-Gricean, and Equilibrium Semantics frameworks, each of which provides partial but incomplete explanations of SI processing. The ERP results contradict the Neo-Gricean Default Model, showing that SI derivation remains effortful even in older children, which challenges the assumption of automatic inference. Prior studies (Nieuwland et al., 2010; Politzer-Ahles et al., 2013) have also reported cognitive cost in SI processing, further challenging the claim of default inference.

The Post-Gricean Pragmatics framework emphasizes context-driven and pragmatic integration. While the findings of this study support the notion that context plays a role in SI derivation, they also reveal that developmental factors significantly mediate implicatures computation. Younger children require greater cognitive resources to compute implicatures, and their ability to process implicatures improves with age. However, traditional Post-Gricean models do not explicitly account for the developmental shift observed in ERP patterns.

The Equilibrium Semantics framework conceptualizes implicature derivation as a game-theoretic equilibrium process, where speakers and listeners strategically adjust meaning. While this model captures the interactive nature of meaning negotiation, it lacks an explicit developmental perspective. The present findings indicate that children do not immediately reach equilibrium but instead exhibit a progressive recalibration of meaning processing. These results align more closely with the CDRM framework, which accommodates both real-time and diachronic changes in SI computation. CDRM diverges from Relevance Theory by positing resource-constrained pragmatic optimization: While adults rapidly compute cost–benefit trade-offs, children’s underdeveloped executive functions necessitate incremental, context-driven SI derivation.

While each of these models contributes valuable insights into implicature processing, they fail to explain how SI competence emerges and evolves throughout childhood. The CDRM bridges this gap by integrating a resource-sensitive, developmentally adaptive perspective that aligns with the observed ERP patterns and behavioral trends.

#### 5.3.1. Integrating a Diachronic-Developmental Perspective

CDRM introduces the concept of diachronic equilibrium, proposing that SI derivation becomes progressively more efficient as children’s cognitive and linguistic abilities mature. This contrasts with Equilibrium Semantics, which assumes immediate balance in meaning negotiation. CDRM accounts for the gradual optimization of implicature computation across developmental stages.

#### 5.3.2. Explaining Resource-Dependent Adaptation

Unlike static models that categorize SI computation as either effortful or automatic, CDRM conceptualizes SI processing as resource-dependent. The ERP findings suggest that implicature derivation is modulated by attentional allocation, semantic memory, and perceptual ability, all of which improve with age. This aligns with research demonstrating that pragmatic inferencing in children is closely linked to their general cognitive abilities.

#### 5.3.3. Providing Neural Evidence for a Multi-Stage SI Processing Model

CDRM is further supported by the multi-stage processing revealed in the ERP data. The distinct contributions of P200, N400, and LPC effects indicate that SI derivation involves multiple cognitive operations, rather than being a single, uniform process. These findings reinforce the argument that scalar implicature computation is a dynamic, developmentally sensitive process that undergoes progressive refinement over time.

## 6. Conclusions

This study employed ERPs to examine SI processing in Chinese preschoolers using CDRM. Findings show that SI derivation in children aged 4–6 is shaped by cognitive development, linguistic ability, and contextual factors. A developmental shift in interpreting “some” occurs by age 5, with rapid progression in pragmatic reasoning by age 6.

CDRM predicts that SI training targeting attentional gating (P200 reduction) accelerates pragmatic mastery, suggesting a potential intervention strategy to enhance pragmatic competence.

While Mandarin N400 latencies (300–500 ms) appear delayed compared to Germanic languages (200–400 ms), direct comparison requires cross-linguistic ERP datasets with matched paradigms. Future work should test CDRM’s cross-linguistic generalizability, particularly in clinical populations (e.g., ASD).

Due to the modest sample size, future studies should prioritize larger cohorts to confirm the critical shift at age 5. Further research should explore a broader range of scalar alternatives, school-aged children, and longitudinal studies to track the developmental trajectory of SI and inform educational interventions.

## Figures and Tables

**Table 1:** Statistical Summary of ERP Component Analysis.

Component	Effect	F-Statistic	*p*-Value	${\eta \rho }^{2}$ (95% CI)	Post Hoc Comparisons	Adjusted *p*-Value
P200	Group × Context Interaction	F(2,45) = 4.577	0.016 *	0.169 [0.06, 0.32]	Lower vs. Higher (Infelicitous)	0.011 → 0.033 *
	Simple Effect (Lower: Fel vs. Infel)	F(1,45) = 7.278	0.010 *	0.139 [0.03, 0.29]	Lower-Felicitous > Lower-infelicitous	0.010 → 0.020 *
	Simple Effect (Lower:Fel vs. Higher)	F(2,45) = 4.980	0.011 *	0.181 [0.07, 0.34]	Lower-infelicitous > Higher-infelicitous	0.011 → 0.033 *
	Simple Effect (Lower:Fel vs. Middle)	-	0.065 *	-	Lower-infelicitous > Middle-infelicitous	0.065 → 0.195 (NS)
N400	Main Effect of Context	F(1,45) = 4.196	0.046 *	0.085 [0.01, 0.22]	Infelicitous > Felicitous (All Groups)	-
LPC	Marginal Main Effect of Context	F(1,45) = 2.956	0.092 *	0.062 [0.00, 0.18]	Infelicitous > Felicitous (Trend)	-

Note: → indicates the adjustment from uncorrected to corrected *p*-values in post-hoc comparisons; NS = not significant (*p* > 0.05); * *p* < 0.05.

## Data Availability

The datasets generated and analyzed during the current study are available from the corresponding author upon reasonable request, subject to ethical and privacy restrictions.

## References

[B1-behavsci-16-00371] Armstrong A., Bulkes N., Tanner D. (2018). Quantificational cues modulate the processing of English subject-verb agreement by native Chinese speakers: An ERP study. Studies in Second Language Acquisition.

[B2-behavsci-16-00371] Bambini V., Arcara G., Martinelli I., Bernini S., Alvisi E., Moro A., Cappa S. F., Ceroni M. (2016). Communication and pragmatic breakdowns in amyotrophic lateral sclerosis patients. Brain and Language.

[B3-behavsci-16-00371] Barner D., Bachrach A. (2010). Inference and exact numerical representation in early language development. Cognitive Psychology.

[B4-behavsci-16-00371] Bates D., Mächler M., Bolker B., Walker S. (2015). Fitting linear mixed-effects models using lme4. Journal of Statistical Software.

[B5-behavsci-16-00371] Bates E. (1976). Language and context: The acquisition of pragmatics.

[B6-behavsci-16-00371] Cheng L., Gao Y., Mao H., Peng Y. (2024). Metapragmatic awareness development in Chinese children: A conversational competence perspective. Journal of Pragmatics.

[B7-behavsci-16-00371] Da Silva F. L., Mulert C., Lemieux L. (2010). EEG-fMRI: Physiological basis, technique, and applications. EEG: Origin and measurement.

[B8-behavsci-16-00371] De Villiers J. G., de Villiers P. A. (1979). Discourse and metalinguistics. Language acquisition.

[B9-behavsci-16-00371] Eiteljoerge S. F., Pouscoulous N., Lieven E. V. (2018). Some pieces are missing: Implicature production in children. Frontiers in Psychology.

[B10-behavsci-16-00371] Fairchild S., Papafragou A. (2021). The role of executive function and theory of mind in pragmatic computations. Cognitive Science.

[B11-behavsci-16-00371] Falkum I. L. (2022). The development of non-literal uses of language: Sense conventions and pragmatic competence. Journal of Pragmatics.

[B12-behavsci-16-00371] Federmeier K. D., Mai H., Kutas M. (2005). Both sides get the point: Hemispheric sensitivities to sentential constraint. Memory & Cognition.

[B13-behavsci-16-00371] Foppolo F., Guasti M. T., Chierchia G. (2012). SIs in child language: Give children a chance. Language Learning and Development.

[B14-behavsci-16-00371] Frank M. C., Goodman N. D. (2012). Predicting pragmatic reasoning in language games. Science.

[B15-behavsci-16-00371] Freunberger R., Klimesch W., Doppelmayr M., Höller Y. (2007). Visual P2 component is related to theta phase locking. Neuroscience Letters.

[B16-behavsci-16-00371] Friedman D., Johnson R. (2000). Event-related potential (ERP) studies of memory encoding and retrieval: A selective review. Microscopy Research and Technique.

[B17-behavsci-16-00371] Hillyard S. A., Münte T. F. (1984). Selective attention to color and location: An analysis with event-related brain potentials. Perception & Psychophysics.

[B18-behavsci-16-00371] Holtgraves T., Kraus B. (2018). Processing SIs in conversational contexts: An ERP study. Journal of Neurolinguistics.

[B19-behavsci-16-00371] Horn L. R., Schriffin D. (1984). Toward a new taxonomy for pragmatic inference: Q-based and R-based implicature. Meaning, form and use in context: Linguistic applications.

[B20-behavsci-16-00371] Horn L. R. (2009). Implicature, truth, and meaning. International Review of Pragmatics.

[B21-behavsci-16-00371] Horowitz A. C., Frank M. C. (2015). Sources of developmental change in pragmatic inferences about scalar terms. Proceedings of the annual meeting of the cognitive science society.

[B22-behavsci-16-00371] Huang Y. T., Snedeker J. (2018). Some inferences still take time: Prosody, predictability, and the speed of SIs. Cognitive Psychology.

[B23-behavsci-16-00371] Jaszczolt K. (2005). Default semantics: Foundations of a compositional theory of acts of communication.

[B24-behavsci-16-00371] Kampa A., Papafragou A. (2023). Children and adults use pragmatic principles to interpret non-linguistic symbols. Journal of Memory and Language.

[B25-behavsci-16-00371] Kandel E. R., Schwartz J. H., Jessell T. M. (2010). Principles of neural science.

[B26-behavsci-16-00371] Katsos N., Bishop D. V. (2011). Pragmatic tolerance: Implications for the acquisition of informativeness and implicature. Cognition.

[B27-behavsci-16-00371] Kutas M., Federmeier K. D. (2011). Thirty years and counting: Finding meaning in the N400 component of the event-related brain potential (ERP). Annual Review of Psychology.

[B28-behavsci-16-00371] Levinson S. C. (2000). Presumptive meanings: The theory of generalized conversational implicature.

[B29-behavsci-16-00371] Li Y. (2004). Child language development.

[B30-behavsci-16-00371] Liang D. (2019). The mysteries of children’s language development. Guangming Daily.

[B31-behavsci-16-00371] Luck S. J., Kappenman E. S. (2011). The Oxford handbook of event-related potential components.

[B32-behavsci-16-00371] Männel C. (2008). The method of event-related brain potentials in the study of cognitive processes: A tutorial. Early language development: Bridging brain and behaviour.

[B33-behavsci-16-00371] Nieuwland M. S., Ditman T., Kuperberg G. R. (2010). On the incrementality of pragmatic processing: An ERP investigation of informativeness and pragmatic abilities. Journal of Memory and Language.

[B34-behavsci-16-00371] Noveck I. A. (2001). When children are more logical than adults: Experimental investigations of SI. Cognition.

[B35-behavsci-16-00371] Noveck I. A., Posada A. (2003). Characterizing the time course of an implicature: An evoked potentials study. Brain and Language.

[B36-behavsci-16-00371] Onton J., Westerfield M., Townsend J., Makeig S. (2006). Imaging human EEG dynamics using independent component analysis. Neuroscience & Biobehavioral Reviews.

[B37-behavsci-16-00371] Panizza D., Onea E. (2014). Some implicatures take their time: An ERP study on scalar implicatures with ‘sentence-picture vs. picture-sentence’ verification task. Talk presented at 27th CUNY conference on human sentence processing.

[B38-behavsci-16-00371] Panizza D., Onea E., Mani N. (2021). Early ERP evidence for children’s and adult’s sensitivity to SIs triggered by existential quantifiers (some). Frontiers in Psychology.

[B39-behavsci-16-00371] Papafragou A., Musolino J. (2003). Scalar implicatures: Experiments at the semantics–pragmatics interface. Cognition.

[B40-behavsci-16-00371] Papafragou A., Tantalou N. (2004). Children’s computation of implicatures. Language Acquisition.

[B41-behavsci-16-00371] Parikh P. (2010). Language and equilibrium.

[B42-behavsci-16-00371] Politzer-Ahles S., Fiorentino R., Jiang X., Zhou X. (2013). Distinct neural correlates for pragmatic and semantic meaning processing: An event-related potential investigation of SI processing using picture-sentence verification. Brain Research.

[B43-behavsci-16-00371] Potts G. F., Patel S. H., Azzam P. N. (2004). Impact of instructed relevance on the visual ERP. International Journal of Psychophysiology.

[B44-behavsci-16-00371] Pouscoulous N., Noveck I. A., Politzer G., Bastide A. (2007). A developmental investigation of processing costs in implicature production. Language Acquisition.

[B45-behavsci-16-00371] Pulvermüller F., Shtyrov Y. (2006). Language outside the focus of attention: The mismatch negativity as a tool for studying higher cognitive processes. Progress in Neurobiology.

[B46-behavsci-16-00371] Röhrig S. (2010). The acquisition of SIs.

[B47-behavsci-16-00371] Sperber D., Wilson D. (1995). Relevance: Communication and cognition.

[B48-behavsci-16-00371] Teresa Guasti M., Chierchia G., Crain S., Foppolo F., Gualmini A., Meroni L. (2005). Why children and adults sometimes (but not always) compute implicatures. Language and Cognitive Processes.

[B49-behavsci-16-00371] Tomasello M. (2018). How children come to understand false beliefs: A shared intentionality account. Proceedings of the National Academy of Sciences of the United States of America.

[B50-behavsci-16-00371] Tomlinson J. M., Gotzner N., Bott L. (2017). Intonation and pragmatic enrichment: How intonation constrains ad hoc scalar inferences. Language and Speech.

[B51-behavsci-16-00371] Van Petten C., Weckerly J., McIsaac H. K., Kutas M. (1997). Working memory capacity dissociates lexical and sentential context effects. Psychological Science.

[B52-behavsci-16-00371] Wilson E., Katsos N., Schneider K., Ifantidou E. (2020). Acquiring implicatures. Developmental and clinical pragmatics.

[B53-behavsci-16-00371] Wu M. (2023). Mandarin children’s scalar implicatures acquisition. Journal of Hubei University of Technology.

[B54-behavsci-16-00371] Yang X., Minai U., Fiorentino R. (2018). Context-sensitivity and individual differences in the derivation of SI. Frontiers in Psychology.

[B55-behavsci-16-00371] Zhao M., Liu T., Chen G., Chen F. (2015). Are SIs automatically processed and different for each individual? A mismatch negativity (MMN) study. Brain Research.

[B56-behavsci-16-00371] Zhao S., Ren J., Frank M. C., Zhou P. (2021). The development of quantity implicatures in Mandarin-speaking children. Language Learning and Development.

